# George Howard Darwin and the “public” interpretation of *The Tides*

**DOI:** 10.1177/00732753231181548

**Published:** 2023-07-18

**Authors:** Edwin D. Rose

**Affiliations:** University of Cambridge, UK; Darwin College, UK

**Keywords:** Public lecturing, book history, material culture, Darwin, geophysics, scientific book, *The Tides*, Lowell Lectures, America

## Abstract

Processes of adapting complex information for broad audiences became a pressing concern by the turn of the twentieth century. Channels of communication ranged from public lectures to printed books designed to serve a social class eager for self-improvement. Through analyzing a course of public lectures given by George Howard Darwin (1845–1912) for the Lowell Institute in Boston and the monograph he based on these, *The Tides and Kindred Phenomena of the Solar System* (1898), this article connects the important practices of public lecturing and book production–two aspects of knowledge dissemination that tend to be studied as separate entities. Darwin, Plumian Professor of Astronomy at the University of Cambridge and son of the famous naturalist, relied on a diverse material culture when lecturing and producing a book. Giving a new account of Darwin’s scientific work through exploring his adaption of it for broader audiences, this article connects the diverse material culture Darwin employed in talks to the practice of producing a published book. The content of objects demonstrated and the lantern slides projected during Darwin’s lectures evolved to form a book designed to engage broad sectors of society in Europe and the United States. Darwin’s lectures were attended at full capacity, while *The Tides* was soon printed in numerous English editions and translated into German, Italian, Hungarian, and Spanish.

On October 13, 1897, the *Boston Herald* announced how “the tide of public interest is just now flowing toward Prof. George Howard Darwin of Cambridge, Eng., who landed in New York last Saturday, and who will arrive in Boston today.”^1^ By the 1890s, Darwin (1845–1912), Plumian Professor of Astronomy at the University of Cambridge, was already a famous name in Britain and the United States. His visit created a surge of interest among the public of Boston. A member of one of the most prominent scientific dynasties of the age and son of the famous naturalist Charles Robert Darwin (1809–82), George Darwin’s reputation emerged from his own research on different forms of oceanic, subterranean, and solar tides, an area of expertise alluded to through the initial metaphor in the *Boston Herald.*^2^

Darwin’s main reason for visiting Boston was to give a course of ten lectures on “The Tides” for the Lowell Institute, an annual series of public lectures founded by a bequest of $250,000 from John Lowell Jr. (1799–1836).^3^ Darwin represents one of the many prominent international Lowell Institute lecturers who visited Boston from the mid nineteenth century, walking in the footsteps of Charles Lyell, Louis Agassiz, Charles Dickens, William Makepeace Thackeray, D’Arcy Wentworth Thompson, Edward Poulton, Alfred Russel Wallace, J. G. Wood, and the Lowndean Professor of Astronomy and Geometry at Cambridge, Robert Ball, who had “paid a high tribute” to Darwin’s achievements.^4^ As Diarmid Finnigan has suggested, many Lowell lecturers spoke on scientific topics and some used the talks as a springboard to embark on subsequent lecture tours of the United States.^5^ The Lowell Lectures attracted large audiences – around 2,000 people in the 1840s, and each lecture continued to attract about 900 people in the 1890s.^6^ These audiences came from a broad range of different professions, although many represented the educated reading public of the period and lectures tended to be constructed with a miscellaneous literate audience in mind. Certain aspects of the content were left open for the audience to interpret.^7^ For many, including Lyell, Wallace, and Darwin, the content of their lectures was either founded on or formed the basis for the content of printed books, emphasizing the close connections between the material aspects of producing a published account and giving a course of lectures for the Lowell Institute.

Although processes of lecturing and producing monographs for broad audiences at the turn of the twentieth century have received attention over the last two decades, studies of public speaking and book production have remained disconnected. For example, Bernard Lightman’s seminal *Victorian Popularisers of Science* has examined the processes of devising public lectures. These remained central for delivering information on the sciences to broad audiences throughout the nineteenth century and Lightman has cast important light on the large number of lecturers who developed approaches for conveying complex ideas to the Victorian public through combining aspects of instruction and entertainment.^8^ Peter Bowler has since successfully argued for the central role of “professional scientists” in producing numerous books aimed at broader audiences, a phenomenon that became widespread in the years around 1900.^9^ Bowler has highlighted the transformative importance of photography when conveying information in published works read by a social class anxious for self-improvement. Different authors’ credentials as academic experts who could communicate their research in everyday language added authority to the content of these publications. Prominent names became important to publishers, who relied on them to generate sales.^10^ George Darwin fitted all these qualifications – his family name alone secured audiences for his lectures and book. Still more recently, Josh Nall has emphasized the process of distributing the content of lectures through newsprint by exploring a series of lectures given by Percival Lowell on Mars in February 1895.^11^ However, it remains the case that limited attention has been paid to the material connections between lectures and the books that emanated from them, developing a set of connected practices in the decades around 1900.

This current study works to unite the practices associated with giving public lectures with the production and sale of a book through examining George Darwin’s Lowell Lectures and the complex process of converting these into his only monograph, *The Tides and Kindred Phenomena of the Solar System* (1898).^12^ It connects a diverse material culture ranging from the temporary exhibitions, objects, and lantern slide projections displayed during public lectures to the practices employed to reproduce these objects, ideas, and images within the pages of a printed book.^13^ Lantern slides are central to these processes, and their crucial role in conveying information has recently been recognized in religious contexts, serving both to instruct and inspire a sense of devotion among broad audiences.^14^ Darwin employed lantern slides to inspire a similar level of awe and devotion in his broad middle-class Bostonian audience, and this article explores the complex processes involved with transferring images projected on the walls of a hall into plates and woodblocks interspersed among the letterpress pages of a monograph. An exploration of these practices casts new light on Darwin’s relationship with John Murray’s publishing house, which had organized the production of many of his father’s works from the 1850s.^15^ Paper remained central to these processes, as it had throughout the lives and careers of members of the Darwin family since the eighteenth century, and plays a prominent role in the process of exploring the stages between giving public talks and producing books.^16^ Images represented in lantern projections regularly moved between glass plates and a variety of published accounts, as did the manuscript notes Darwin relied on when lecturing. These words later found themselves set in letterpress and printed in books and articles to surround woodblock and electrotype prints that originated from lantern projections.

An examination of the Lowell Lectures given by George Darwin in the autumn of 1897 and his subsequent book *The Tides* shows how complex scientific theories were transformed and solidified as printed works for broader audiences. After giving a brief introduction to Darwin’s academic work, the initial sections explore his approaches to giving lectures while integrating projected images and demonstrations of scientific instruments to engage his audience. The next section moves onto the processes behind transferring an oral presentation and associated objects and images into a printed book to uncover the complex relationships Darwin developed with his publisher, scientific illustrator, and printer. The final sections conclude by assessing the reception of Darwin’s *Tides*, exploring its numerous editions and translations, the practices Darwin used to convey a set of specific talks to a global audience, and the legacy of this work that extended into the twentieth century.

## Recruiting a speaker

The house of Newnham Grange in central Cambridge, the home of George Darwin’s family from 1885, regularly received letters from across the globe.^17^ These came from Africa, India, the Americas, and Australia and included responses to Darwin’s many questions on tidal and astronomical movements. Those from the United States always included a mixture of academic and personal correspondence addressed to both Darwin and his wife, Maud du Puy (1861–1947), herself a member of the Du Puy family from Philadelphia and a prominent figure in Cambridge society.^18^ In May 1896, George Darwin received a letter from the wealthy New England industrialist Augustus Lowell (1830–1900), the principal trustee of the Lowell Institute since 1881, who was responsible for inviting speakers to give lectures.^19^ Lowell wrote “to ask if it would be possible for you to come to this country next winter 1896–1897 to deliver a course of lectures upon the Tides for the Lowell Institute of this City. Two Lectures are expected each week of an hour’s length at $150 or £30 a lecture.”^20^ A week after receiving Lowell’s letter, Darwin drafted his response. Writing on May 10, 1896, Darwin declined Lowell’s offer, stating that “the preparation of popular lectures on so technical a subject as the tides would take a considerable amount of time. . .it would be December before I could seriously attack the work.” However, rather than turning down Lowell’s offer altogether, Darwin asked if he could give the lectures in 1897 to “give me the longest notice possible.”^21^

Unlike his famous father, the naturalist Charles Robert Darwin (1809–82), whose various evolutionary theories had caused ripples of controversy throughout nineteenth-century society, George Darwin presented the perfect candidate for a lecturer at the Lowell Institute. Darwin’s detailed mathematical analyses of the tides fitted with the specifications laid out in John Lowell’s will, which stipulated that lectures at the institute should be given while “not engaging in controversy.”^22^ Although not controversial, Darwin’s distinct interdisciplinary interests were central for shaping his research. Throughout his life, Darwin rejected the term “scientist” as a “new and mongrel word,” believing it was too limiting when it came to the exploration of the natural world and his own interests in combining the fields of geology, mathematics, and astronomy to found the field of geophysics.^23^Since moving to Trinity College, Cambridge, in 1868, Darwin had developed interests in the shape, movement, and physical makeup of the earth. His first article on the subject, entitled “On the Influence of Geological Changes on the Earth’s Axis of Rotation,” built on the work of William Thompson, Lord Kelvin, through assessing that the angle between the earth’s equator and its orbit remained relatively constant throughout deep time, suggesting the earth exhibited a plasticity by making rough readjustments to a figure of equilibrium, causing the pole to move about ten or fifteen degrees.^24^ In comparison, Thompson theorized that the earth was an elastic solid. Darwin adapted Thompson’s hypothesis to show that similar results could be achieved by treating the earth as viscous fluid, although he was careful to note that “It will of course be understood that I do not conceive the earth to be really a homogeneous viscous or elastico-viscous spheroid, but it does seem probable that the earth still possesses some plasticity.”^25^ It was Thompson who reviewed this paper and, as a result of Darwin’s mathematical talent, wrote a sixteen-page report praising the research. Soon after, Thompson invited Darwin to Glasgow, an initial discussion that stimulated the production of a whole series of papers that elevated Darwin into the scientific elite.^26^

Darwin’s interests expanded into oceanic tides in the late 1870s, when he began to commission Cambridge University Press to print tide timetables. In 1891, these cost £10/10s per forty copies and were distributed, with assistance from the British Admiralty, to hydrographic offices across the world.^27^ Printed tables were then filled out and sent back to Darwin from localities including Aden, Batavia, Puna, Hong Kong, Adelaide, and Cape Town, sending records charting daily changes in the tides over several decades to support Darwin’s belief that tides could only be understood through systematic observation.^28^ As a result, Darwin quickly became, in David Kushner’s words, “the government clearing house for the organisation and reduction of tidal observations across the British empire.”^29^ By 1896, Darwin required additional assistance with the volume of calculations, receiving a Royal Society grant of £100 “for payment of computers” – highly talented practical mathematicians whose extensive correspondence fills a significant portion of Darwin’s archive.^30^ From the late 1880s, Darwin began to promote this research to broad audiences by giving individual public lectures and publishing illustrated adaptations of these talks in magazines. This developed Darwin’s reputation as an engaging public speaker, making him an ideal candidate for a course of lectures at the Lowell Institute.

## Anticipating Darwin’s lectures

After agreeing to give a series of ten lectures entitled “Tides” in Boston, Darwin enquired into the Lowell Institute’s preferred approaches to presenting. Writing to Darwin on March 4, 1897, Lowell outlined the process of giving lectures and enclosed a seating plan of the Huntington Hall, part of the Massachusetts Institute of Technology (MIT). In addition to outlining the precise room used to give lectures, Lowell noted:

You will see by the enclosed notice that Stereopticon views (not diagrams) are to be shown at the end of the lecture which was not meant to apply so far as they were needed in illustration of the text, but avoid their too liberal use unless the audience were willing to remain beyond the hour for the pleasure of seeing them.^31^

**Figure 1.**
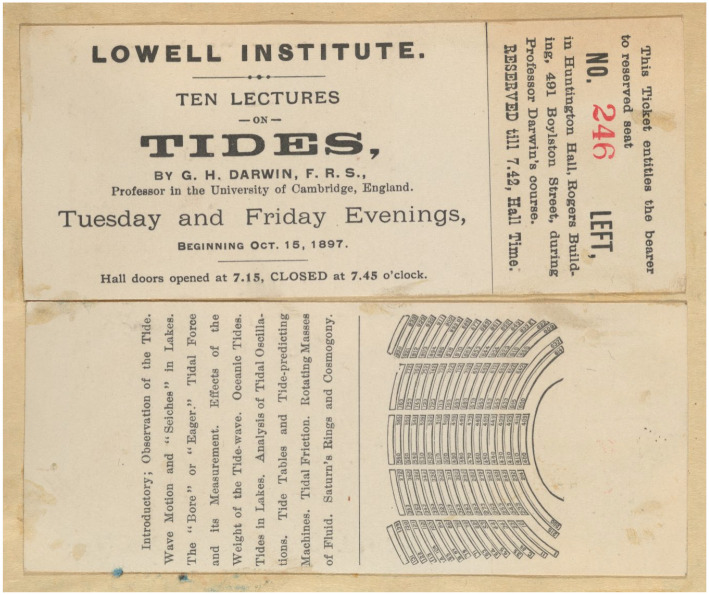
An entrance ticket and seating plan for George Darwin’s Lowell Lectures on “Tides” in 1897. 54M-206, Houghton Library, Harvard University.

According to the printed notice outlining the proportions of the hall and the specific timings for each lecture, the Huntington Hall contained “ample space” for the “display of maps, diagrams etc.” while “when necessary the Institute provides a stereopticon, but the lecturer should furnish the requisite ‘slides.’”^32^ Lowell’s communication of a plan of the room to Darwin, identical to that shown on the printed ticket in Figure 1, was essential for allowing Darwin to plan a course of lectures that utilized both slides and demonstrations of experiments from the offset. This presents similarities with figures such as John Tyndall (1820–93), who, when giving Lowell Lectures on light and heat in 1872–3, worried that the large American audiences might not be able to see the equipment across the spacious auditorium and had to find a way to ensure audiences could view his experiments.^33^ Projection was critical to Tyndall’s Lowell Lectures in 1872: it ensured the audience could view enlarged images of the experiments through a variety of lenses and “a magic lantern strongly supported on a platform with wires attached indicating electricity as the source of light.”^34^ It seems that Darwin used similar, if not the same, equipment in 1897. Experiments were typically performed during lectures and integrated with the showing of lantern slides that the institute’s bylaws stipulated “should be given at the close of the lecture.”^35^ Rules such as this were most likely a legacy of speakers from the 1870s, who used so many slides it proved to be burdensome for the Lowell Institute’s staff.^36^ Each talk was designed to be an hour in length and “written, or prepared, for the Lowell Institute” and not given elsewhere in Boston before the specified lecture.^37^

For Darwin, the ability to show slides in lectures was central for conveying complex research to broad audiences. Writing to Lowell on March 17, 1897, Darwin asked, “if you could let me know the size of the slides for the magic lantern, if I should desire to exhibit any of my diagrams in that way.”^38^ Darwin quickly realized that rather than showing projected images at the end of lectures, these needed to be integrated into the main body of the talk. As such, Darwin disagreed with the printed notice, requesting to show slides during his talk, since “I shall require them to explain what I am saying.”^39^ This reflects the importance of engaging an audience through showing a range of images to support the main arguments and ideas given in a popular lecture, and the limited number of slides Darwin proposed to show resulted in the rule of only showing slides at the end being relaxed.^40^ For example, Robert Ball (1840–1913), Darwin’s colleague at Cambridge, had been criticized after giving a course of Lowell Lectures in 1884. The *Boston Herald* noted that Ball had an “impediment in his speech,” while describing his lecturing style as “clergymanic.”^41^ As Lightman has suggested, by the 1880s and 1890s audiences attended public lectures to be instructed and entertained, and it was the responsibility of a lecturer to provide this service.^42^ Images, portable exhibitions, and displays formed a key part in this process and had central roles in Darwin’s course of Lowell Lectures.

Widescale interest in Darwin’s lectures was made clear by the *Boston Herald*, which described Darwin as “a successful explorer in an unknown region of science and [who] has brought back facts of real importance.” It was also suggested that “a certain dramatic interest is added to his career from the fact that his father was Charles Darwin, the originator of the Darwinian theory of the origin of species.” In addition to emblazoning an article entitled “Professor Darwin Comes Today” with a profile illustration of Darwin, the *Herald* outlined the popularity of his lectures before they even commenced: “every seat at his [Darwin’s] lectures is spoken for in advance and somewhere in the neighbourhood of 900 people will be gathered on Friday evening to hear him.”^43^ Attendees were presented with a Lowell Institute ticket, outlining a seat number and a plan of the hall on the verso (Figure 1).^44^ Darwin’s course commenced on Friday, October 15, 1897, and each subsequent session took place on Tuesday and Friday evenings. There was no tail-off in attendance and the *Herald* described how Darwin’s series was “one of the largest gatherings ever attracted to a purely scientific Lowell Institute course.”^45^

When it came to his lecturing style, Darwin almost certainly drew on his previous experience. Much of this came from his teaching in Cambridge, giving the Bakerian Lecture for the Royal Society in 1891, and his expanding portfolio of public lectures that he gave for the Royal Institution, the Birmingham and Midland Institute, the Gilchrist Trust, and the Sunday Lecture Society in the 1880s and 1890s.^46^ Darwin also received advice from Ball, who had an extensive knowledge of how to entertain and engage diverse audiences and experience of delivering a course of Lowell Lectures.^47^ Interest in entertaining the audience encouraged Darwin to combine his use of lantern slides with an animated presentation style, a practice alluded to in the *Boston Herald*:

Prof. Darwin went on to show his audience, with the aid of diagrams, how tides are raised on the surface of the earth by the attraction of the sun and moon. The exposition, though mathematical in character, was given in simple language, and with such intelligibility as to elicit the hearty applause of the audience.^48^

Lecturing alongside projected images retained Darwin’s large audience, while the illustrative material served to define each session. One journalist described Darwin’s practice of giving lectures: “The Professor uses manuscript, and is most unostentatious in manner and speech.”^49^ Subjects Darwin covered included “Observation of the Tide-Wave Motion and ‘Selches’ in lakes. The ‘Bore’ or ‘Eager.’ Tidal force and its Measurement. Effects of the Weight of the Tide-Wave. Oceanic Tides. Tides in Lakes. Analysis of Tidal Oscillations. Tide Friction. Rotating Masses of Fluid. Saturn’s Rings and Cosmogony.”^50^ Darwin later broke down the two themes of each lecture into twenty chapters reproduced in *The Tides*.

## Projecting pictures and presenting objects

When preparing for his Lowell Lectures, Darwin commissioned a series of lantern slides before departing from England. It is probable that some were produced in Cambridge by firms that manufactured lantern slides for scientific purposes or to depict scenes shown at evening parties in middle-class homes. By the 1890s, scientific lantern slides became easy to obtain. Many British scientific societies integrated the use of the lantern as a standard means for conveying information in their academic and public lectures. This approach was encouraged at the Royal Geographical Society by figures such as Francis Galton, a cousin of Darwin’s father, who stimulated the Society’s gradual adoption and integration of the magic lantern into its lectures.^51^ The Royal Astronomical Society played an active role in supplying slides to lecturers. Examples include those Ball purchased for one shilling each during the 1880s and 1890s for use on popular lecture tours.^52^

In comparison to Ball, Darwin’s slides are private commissions designed to sustain the engagement of the audience. This is apparent from the specificity of the images they contain, ranging from photographic reproductions to mathematical diagrams.^53^ Darwin’s first series of photographic slides were shown in his second Lowell Lecture covering tidal bores. These images are based on a series of photographs taken by Captain William Usborne Moore (1849–1918) in 1888 and 1892 when undertaking surveying missions in the estuary of the Tsian-Tang-Kiang, also referred to as Qiantang, near Hangzhou in Eastern China.^54^ The British recognized the importance of establishing a naval presence in this estuary, an essential maritime artery for accessing export markets in the Chinese interior.^55^

**Figure 2.**
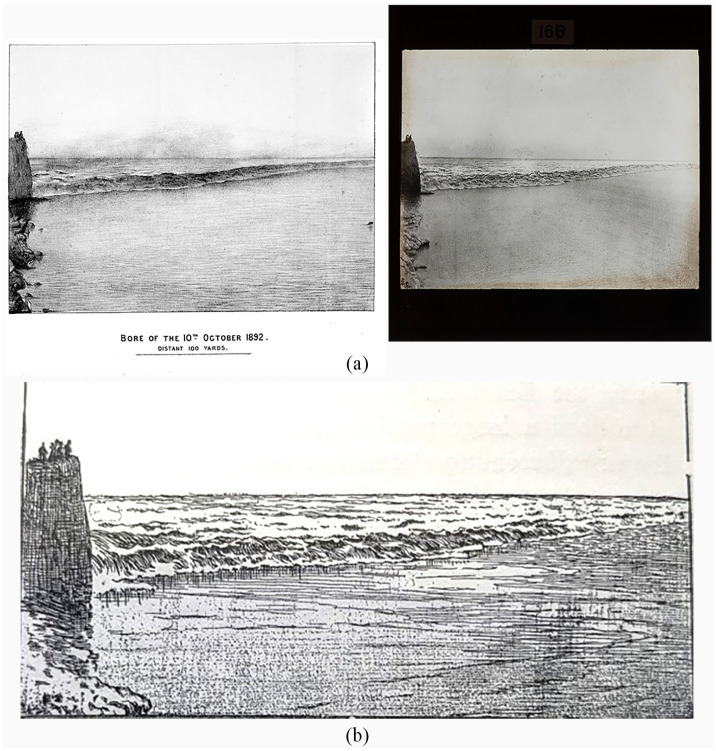
(a) (Left) The photograph published in Moore’s *Further Report on the Bore of the Tsien-Tang-Kiang* (1893). (Right) The lantern slide Darwin showed in Boston in 1897. Reproduced with the kind permission of the Master and Fellows of Darwin College, Cambridge. (b) One of a series of woodcut illustrations depicting the bore on the Tsian-Tian-Kiang published in Darwin’s *Tides and Kindred Phenomena of the Solar System*.

The photographs Darwin projected were taken by Moore in 1892 when commanding a squadron of British ships led by HMS *Penguin*. These images came to Darwin’s attention after he read Moore’s reports of the expedition, published in limited edition pamphlets by the British Hydrographic Office after each surveying mission.^56^ Darwin received these reports through his links with figures such as Admiral William Wharton (1843–1905) at the Hydrographic Office, who supplied him with data and facilitated the distribution of his tide tables to hydrographic offices across the British empire.^57^ Moore also published several photographs that Darwin had reproduced as lantern slides. These were designed to present a spectacle for Darwin’s audience in Boston, depicting the full force of the largest tidal bore in the world. For example, the image showing the bore passing a headland on October 10, 1892, is a direct reproduction from Moore’s pamphlet (Figure 2a). Darwin’s next slide shows comparative images of the bore at full force and one minute later where the water is still rough. In the caption, Darwin relied on Moore’s account to describe the specific positioning of the “Camera established 20 feet above the River, behind the buttress west of the Pagoda at Hai ning.”^58^ The bore caused considerable damage to Moore’s squadron, forcing two of the ships to drag “their anchors three miles up the river.”^59^

Darwin gave a simplified account of the lunar origins of tidal bores and the stimulation of the wave through the specific conditions of the depth and shape of the river. However, in the monograph based on his Lowell Lectures, Darwin assessed that “the complete theory of waves would be too technical” for such lectures, giving the rest of his talk over to the dramatic events Moore’s squadron experienced, historical accounts of the bore, and his assessment of the proposal to harness tidal bores as a renewable energy source.^60^ Darwin wrote, “it has been supposed by many that when the coal supply of the world has been exhausted we shall fall back on the tides to do our work,” summarizing the power of these natural events and their potential for solving the problems of contemporary society. This contrasts with the opinions reported during Darwin’s Lowell Institute course. For example, the *Boston Evening Journal* published an article entitled “Can’t be done: Prof. Darwin says that tides are not available for generating power.” After describing how Darwin’s audiences “are among the largest ever seen at these lectures,” the newspaper emphasizes Darwin’s comments on the inefficiencies in harnessing the tides as a source of power, “since the energy of the tides, boundless as it is, does not exist in a form which is easily available.”^61^ This mirrors Darwin’s comments in his later monograph, in which he described the inefficiencies of contemporary technology for harnessing this power, emphasizing that tidal energy can only be harnessed in specific places such as tide mills in river estuaries. Darwin’s analysis of the tides’ potential use as an energy source was utilized by J. B. S. Haldane in *The Last Judgement: A Scientist’s Vision of the Future of Man* (1927), who outlined that “It is suggested that the effect of tidal friction in slowing down the earth’s rotation and therefore lengthening the day, was first discovered by George Darwin, a son of Charles Darwin who gave the first satisfactory account of evolution.”^62^ Haldane theorized that the continual use of tidal power would slow the rotation of the earth, resulting in mass extinction of non-domesticated species, climate change, and the diminution of the human population.^63^

Moore’s photos and account of the tidal bore on the Tsian-Tang-Kiang led Darwin to describe how “the Chinese regard the bore with superstitious reverence,” reiterating a legend concerning how the bore originated from the spirit of a general whose body, after his assassination on the orders of the Chinese Emperor, was thrown into the Tsien-Tang-Kiang, where the vengeful spirit conceived the idea of bringing the tide in from the ocean, destroying Hangchow, at this time the Chinese imperial capital.^64^ Darwin valued this information, suggesting that “this story is remarkable in that it refers to the reign of an Emperor whose historical existence is undoubted. It thus differs from many of the mythical stories which have been invented by primitive peoples to explain natural phenomena.”^65^ To retain the attention of his large audience, Darwin went on to describe the various social customs surrounding the bore: “The people of Haining still continue to pay religious reverence to the bore, and on one of the days when Captain Moore was making observations some five or six thousand people assembled on the river wall to propitiate the god of the waters by throwing in offerings.”^66^

Shared interests in tidal bores and work for the Hydrographic Office placed Moore on Darwin’s radar, initiating their correspondence. However, Moore’s account of the tidal bore on the Tsian-Tang-Kiang, like many government reports, was a rare publication printed on behalf of the British Admiralty and only distributed to hydrographic offices, institutions, and individuals such as Darwin.^67^ Darwin’s Lowell Lectures are the first attempt to convey this information to a public audience. To make the bore in China more relatable, Darwin describes his attempts to see the bore on the River Severn in England, outlining how “In September 1897 I was on the banks of the Severn at spring tide; but there was no proper bore, and only a succession of waves up stream and a rapid rise of water-level.”^68^ Such was Darwin’s obsession with tidal bores, he timed visits alongside family holidays. His daughter, Margaret Keynes, described in her diary their visit to see the bore on the estuary of the Seine in 1904: “a great wave dashing over the quay. A great many people got wet – one man in a thick motoring coat was taken right off his legs.”^69^

In addition to photographic slides, Darwin relied on diagrams and apparatus to convey complex ideas. Writing to the janitor of the Huntington Hall toward the end of his lectures on November 21, 1897, Darwin gave permission “to allow Professor B. O. Pierce of Harvard University to remove my case of models.”^70^ In comparison to earlier lecturers such as John Tyndall, Darwin’s smaller audience made using models more feasible, ensuring they played a central role in simplifying the mathematical content of Darwin’s lectures. For example, the *Boston Herald* described how Darwin “showed with the aid of diagrams that the frictional retardation of the earth’s revolution by the action of tides is to lengthen the period of the rotation of the earth, and at the same time to lengthen both the day and the month.”^71^ This relates to Darwin’s prominent use of scientific instruments in lectures that discussed tides within the body of the earth. When describing the deflection of the vertical, Darwin described how he and his brother, Horace Darwin (1851–1928), the head of the Cambridge Scientific Instruments Company, had developed a bifilar pendulum to measure ultra-microscopic displacements of the ground.^72^ This was one of several objects Darwin used in his Lowell Lectures both to prove his argument and provide entertainment for the audience. The *Boston Herald* described Darwin’s use of this scientific equipment:

To his large audience he gave an interesting account of methods which have been adopted for measuring the tide-generating force of the moon, among them being a bifilar pendulum, constructed by himself and his brother, Horace Darwin. Extraordinary precautions had to be taken to protect this instrument from local sources of tremor. Its movements in a line north and south were magnified by means of a rotating mirror, attached to the pendulum, the results reached were mainly negative.^73^

**Figure 3.**
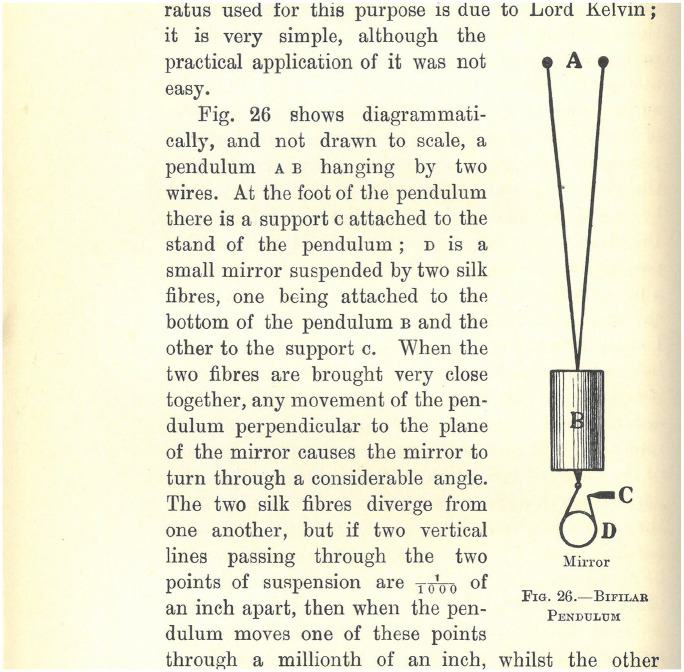
A woodcut diagram depicting Horace Darwin’s bifilar pendulum that George Darwin demonstrated during his Lowell Lectures. This image was first published in *The Tides* (1898) and printed in all subsequent editions.

This allowed the large audience attending Darwin’s Lowell Lectures to gain firsthand experience of the processes of gathering the data used in Darwin’s mathematical calculations while proving the existence of viscous tides in the body of the earth. Such was the importance of showing this object that Darwin later commissioned a woodcut for *The Tides* in which he gives a detailed description of the process of using this equipment (Figure 3).^74^ Although Darwin’s attempts to use the bifilar pendulum in Boston proved unsuccessful due to background interference with the measurements, he described how he used it in secure laboratory conditions in Cambridge, proving that, once all background noise had been eliminated, “it remains certain that a large proportion of these mysterious movements are due to minute earthquakes.”^75^ Darwin also intended for the bifilar pendulum to measure the action of the moon, a usable function after his brother, Horace, perfected the apparatus in the early years of the twentieth century.^76^

Many of the terrestrial events Darwin described, depicted on slides, and demonstrated with instruments were interconnected with the wider solar system and universe. Similar to the tidal bore, Darwin relied on photographic lantern slides to give the public of Boston some of the earliest views of deep space. Typical examples include the slide Darwin showed that depicts the “Nebula in Andromeda,” a photo taken by the noted Liverpool-based amateur astronomical photographer Isaac Roberts (1829–1904) on 1 October 1888 who sent Darwin the slide on 29 December 1888.^77^ However, rather than reproducing a printed illustration, Darwin obtained the slide depicting Andromeda directly from Roberts. Darwin reused this slide in Boston; he acquired it in 1888 when preparing a lecture entitled “On Meteorites, and the History of Stellar Systems” that he gave at the Royal Institution on January 25, 1889.^78^ One audience member described how Darwin used Roberts’s image while “giving a lecture at the Royal Institution on the Mechanical Conditions in a swarm of meteorites,” in which it was suggested the image embodied “the main ideas of the Nebula Hypothesis.”^79^ Similar images became increasingly commonplace in late nineteenth-century astronomical accounts, although it is important to emphasize that contemporary interpretations of Andromeda were very different to the current conception of a galaxy.^80^ Writing to Darwin on January 29, 1889, Arthur Nichols, Organizing Secretary of the Sunday Lecturing Society, asked from where he might procure Darwin’s images, adding that he would “always acknowledge any permission that may be given to make use of illustrations in lecturing.”^81^ In his response, Darwin noted that the images were the product of his extensive correspondence with Roberts, to whom Nichols resolved to write for permission to use the images in further public lectures.

**Figure 4.**
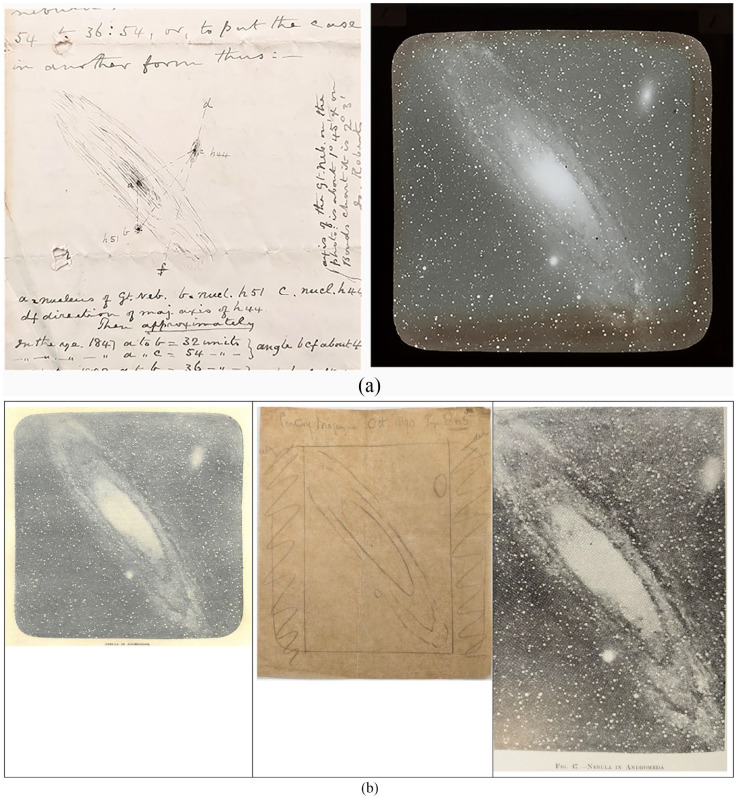
(a) (Left) Isaac Roberts’s letter to Darwin, dated December 4, 1888, in which he sketched Andromeda and outlined the angles of the smaller nebulae. DAR 251: 3379, reproduced with the kind permission of the Syndics of Cambridge University Library. (Right) the lantern slide transparency Roberts sent Darwin in January 1889, entitled “The Great Nebula in Andromeda.” Reproduced with the kind permission of the Master and Fellows of Darwin College, Cambridge. (b) (Left) An image of the Nebula in Andromeda that Darwin published in the *Century Magazine* in 1890. Centre: Edwin Wilson’s illustration showing the process of cutting the electrotype plate and image down from the *Century Magazine* for *The Tides* (1898). Reproduced by kind permission of The Trustees of the National Library of Scotland. (Right) The revised illustration published in Darwin’s *Tides*.

Darwin started corresponding with Roberts in late 1888 and the chief topic of their correspondence was the supply of lantern slides for public lectures. Writing to Darwin, Roberts noted “that you wish to have a lantern transparency of the neb. in Andromeda for a lecture next month – I will send you one before the end of this month and in the mean time hope for 3 or 4 hours of clear sky to enable me to obtain a better negative than the present one.”^82^ In addition, Roberts produced a sketch of Andromeda on the verso of his letter so Darwin knew what to expect (Figure 4). In comparison to earlier depictions, Roberts suggested that “the two nebulae, one on each side of the minor axis of the Great Nebulae known as p44 & p45,” had changed since their original observation by George P. Bond “to the extent of about 25° since the year 1847 or else Bond has wrongly drawn it.”^83^ Roberts’s image and description gave Darwin the opportunity to update the lecture he was preparing for the Royal Institution with this crucial data before the new lantern slide arrived, information combined with an image Roberts believed “to demonstrate the truth of the Nebular Hypothesis.”^84^

Roberts’s lantern slide depicting Andromeda arrived in time for Darwin to give his lecture at the Royal Institution, although, owing to cloudy skies, Roberts had to send “a transparency of the photo taken on 1^st^ October” 1888. Roberts was only able to take photos of the nebulae infrequently due to the cloudy weather – before 1888, the last time he was able to photograph Andromeda and the other nebulae was in 1886. Roberts maintained that photographs “will convey to you more information than would pages of written descriptions” – a belief Darwin maintained when presenting on the nebulae hypothesis before public audiences.^85^

Lantern slides formed a crucial component for Darwin when lecturing on the nebulae hypothesis in the decade before he gave the Lowell Lectures in Boston. In addition to lecturing at the Royal Institution in 1889, Darwin used these slides for numerous public talks across Britain. Darwin intended to publish versions of these lectures and associated slides, the importance of which he emphasized in a letter to the American poet and editor Richard Watson Gilder (1844–1909) when requesting to publish an article in the *Century Magazine*: “The illustration in question is a very extraordinary photograph made by Mr. Roberts of Liverpool of the nebula in Andromeda. This picture appears to me to prove the substantial truth of Laplace’s Nebular hypothesis. As that hypothesis is a step in my lecture this picture is of importance.”^86^ By the time of Darwin’s Lowell Lectures in 1897, recent developments in the field of celestial systems meant that he used this slide to outline a very different account of Laplace’s theory. Laplace’s nebulae hypothesis suggested that the matter that now forms the solar system once existed as a lens-shaped nebula of highly rarefied gas that followed the contours of the current solar system. Initially expanded by heat, Laplace suggested the central section condensed with increased rotation.^87^

When in Boston, Darwin used the slide showing Roberts’s image of the Andromeda nebula to draw attention to potential flaws in Laplace’s theory. Although Darwin acknowledged that some believed Roberts’s image showed “Laplace’s lenticular nebula with its central condensation,” Darwin noted how “other observers, on the other hand, regard it as a spiral nebula seen almost edgewise; and if they are right its formation can hardly be explained according to Laplace’s scheme.”^88^ The shape of Andromeda led Darwin to propose a series of challenges to Laplace’s theory, suggesting “that every stage in the supposed process presents to us some difficulty or impossibility. Thus we ask whether a mass of gas of almost inconceivable tenuity can really rotate all in one piece, and whether it be more probable that there would be a central whirlpool surrounded by more slowly moving stars.” Photographs remained a central reference point for Darwin’s critique of Laplace’s nebulae hypothesis, ideas that developed as a direct result of his public lectures and subsequent publications, adding that “he has been put into possession of countless accurate pictures of nebulae.”^89^ Roberts remained Darwin’s main source of astronomical images. In January 1889 he wrote to Darwin to say he was “making a presentable enlarged transparency for the Lantern, of the Dumbbell neb. & inform you of the result.”^90^ The very different shape of the Dumbbell Nebula encouraged Darwin to exercise caution and not completely discount Laplace’s theory, suggesting that among the infinite number of nebulae scattered across deep space, “it is reasonable to suppose that amongst them must be found representatives of the type of nebula from which the solar system sprang.”^91^

Photographs remained a central means for conveying information on deep space to the broad audiences who attended Darwin’s lectures. This had become evident to Darwin from 1887, when Paxton Porter, secretary of the Birmingham and Midland Institute, suggested “that other lecturers on astronomical subjects have found Lantern slides to be the most useful in our theatre.”^92^ Darwin gave several lectures in Birmingham from the late 1880s, and the organizers of these events were already aware of the importance of showing lantern slides if speakers were to engage their broad middle-class audience. After showing Roberts’s image at the Royal Institution in 1889, Darwin received regular requests for its reuse by public lecturers. Writing to Darwin from Hampstead, Charles Edward Reade described how “I should so much like to have a copy of the nebula in Andromeda by Mr. Roberts,” since it conveyed hitherto unseen structures.^93^ Therefore, Darwin continued to have Roberts’s image projected in his talks, including the Lowell Lectures in 1897. It also formed a central component in all his popular publications. Writing from the editorial department of the *Century Magazine*, Gilder noted his enthusiasm for reproducing such a photograph so long as they are “not engraved and published in any book or periodical elsewhere before they are engraved and published by us.”^94^ Roberts continued to allow Darwin to publish his photographs since Darwin was a route to social elevation in scientific circles. Roberts wanted his images to be distributed on a global scale, commenting that Darwin was “quite at liberty to publish any of my photos & if it will help you I will with pleasure make you a special enlargement to any scale you may desire of those you want.”^95^

## Preparing to publish

Writing to Lowell on March 17, 1897, Darwin stated that “I presume that short-hand writers are not permitted in the hall, so that I shall retain copyright in my lectures in case I should come to publish them as a book.”^96^ Although he had already given numerous public lectures and published many of these as popular articles in periodicals such as the *Century Magazine* and *Harper’s Magazine*, Darwin was already planning to publish the content of his Lowell Lectures after their enthusiastic reception in Boston.^97^ However, Darwin had to make significant adaptions to the published version, commenting in the preface that “This book contains the substance of what I then said. The personal form of address appropriate for a lecture is, I think, apt to be rather tiresome in a book, and I have therefore taken pains to eliminate all traces of the lecture from what I have written.”^98^ This shows that Darwin undertook substantial edits when converting his talks into the content for a book. Other Lowell Institute lecturers, such as Alfred Russel Wallace, who gave a Lowell Institute course in 1886, took a similar approach, basing the main chapters of his book *Darwinism* (1889) on his lectures while the content of the printed version was heavily worked up from the spoken performance.^99^ It seems that Darwin was preparing samples of his manuscript for publication while in Boston. Writing from his premises at 50 Albemarle Street, London, Darwin’s chosen publisher, John Murray, noted that:

I have read the chapter which you kindly submitted to us yesterday with much interest. The subject is a very curious and attractive one, and I cannot but believe that a popular treatise on it will have a good prospect and a favourable reception especially when it has the advantage of your name as author.^100^

Darwin had been familiar with the workings of Murray’s firm since his father’s death in 1882, when he wrote “to obtain for probate a valuation of my father’s books, and generally to know the conditions of your business relations with my father.”^101^ Since the early 1880s, Darwin had maintained a strong business relationship with Murray, acting as a main point of contact in the Darwin family and managing the royalties for his father’s works and the numerous new editions. Among the most important was the 1901 edition of *The Origin of Species*, which had a far greater global impact when compared to earlier editions.^102^

Images remained essential for Darwin when communicating complex ideas to broad audiences in print – just as they did in public lectures. *The Tides* contained numerous illustrations made up from a mixture of woodblocks for diagrams and electrotype plates for complex photographic reproductions. Darwin already had extensive experience of producing images for scientific books. During the 1870s, he had produced illustrations for his father’s book *The Movements and Habits of Climbing Plants* (1875).^103^ Before 1897, Darwin maintained reservations about the quality of illustrations produced by British publishers and the content of the publications in which they appeared. Writing to Gilder about publishing in the *Century Magazine*, Darwin noted: “I do not much think of sending it to an English magazine, because I want a good woodcut & only the lighter English magazines have illustrations.”^104^ Thus, when preparing *The Tides*, Darwin explained to Murray that:

There will be a considerable number [of images] in the first three chapters & much fewer thereafter. The nature of the text will to some extent depend on the manner in which they are reproduced. I think I can get them done most satisfactorily under my own eyes, and therefore it would be a considerable convenience & advantage to have them done here. Mr. Edwin Wilson is a first rate man & does many illustrations for Scientific journals. He has also done all my diagrams for my lectures. In a good many cases a photographic reduction of the diagram will be best & I should like to go to him and discuss with him how each block is to be done, so as to decide whether photography or redrawing would be best.^105^

Darwin’s employment of a specialist scientific illustrator remained a crucial means for him to retain control over the production of *The Tides* and ensure the images conveyed information that aligned with the theories discussed in the surrounding text.

Wilson’s ability to produce illustrations was already well known to many Cambridge professors, the University Press, and the Philosophical Society, of which Darwin was president in both 1890 and 1910.^106^ By the 1890s, Cambridge University Press regularly commissioned Wilson to produce wood engravings and lithographs, following the increasing assumptions that academic texts should contain images that followed on from the increased prevalence of images in magazines.^107^ Authors in both the arts and sciences relied on Wilson’s images. Examples include M. R. James (1862–1936), who commissioned Wilson to produce the plates accompanying a catalogue of manuscripts in Trinity College. Similarly, Cambridge University Press employed Wilson to produce woodblocks for Charles Godfrey’s *Elementary Geometry* (1903), and by 1913 had become so reliant on images that they purchased Wilson’s business.^108^ For Darwin, extensive experience in producing scientific images made Wilson the obvious choice to produce images for *The Tides*. This was coupled with Wilson’s close geographical proximity: it seems Wilson’s business was based on Mill Lane in central Cambridge, close to the main offices for Cambridge University Press. Darwin’s home at Newnham Grange was within easy walking distance.^109^ A close geographical proximity meant Darwin could monitor the quality of the images and avoid problems in the later production process.

Since *The Tides* was designed for a broad array of middle-class readers who sought to extend their understanding of the world around them, many of the images Darwin commissioned were not his own original creations or commissions. For example, the images of the tidal bore were obtained from Moore’s article and the picture of the Andromeda Nebula was taken by Isaac Roberts, although Darwin remained the first person to present this image both as a lantern slide and in a published account.^110^ Darwin had already reproduced several of these illustrations in magazine articles, and both Roberts and Moore had given him express permission to reproduce their photographs. Other images were sourced from recent publications. When writing to Murray about printing mathematical diagrams, Darwin suggested, “There are several woodblocks which may probably be borrowed from Taylor & Francis (if not destroyed). They were used in Baird’s Manual of Tidal Observation. These blocks were on p. 6, p. 3, p. 8 of that book. As I know Col. Baird I am sure that he will give his consent if he knows it is for me.”^111^ Darwin’s extensive network was responsible for supplying many of the images for *The Tides*. Contributors, including Moore and Roberts, saw Darwin’s citation and reproduction of their images as increasing their circulation, elevating their social standing in scientific circles (Figure 2b).

In the end, Wilson produced twenty-four illustrations for Darwin’s book, at a total cost of £19/10s, in addition to securing the other blocks from Taylor and Francis, since, as Darwin commented to Murray, he had “a good deal to do with them before.”^112^ Images in *The Tides* were often set within letterpress that Darwin designed to be read in conjunction with the illustrations. This necessitated an accessible font, with Darwin outlining to Murray that “You may remember that you sent me a sample page as a suggestion for size & Type. I rather prefer the type of my brother’s book, to that of the sample; but the difference is only slight.”^113^ This comment relates to Leonard Darwin’s (1850–1943) popular treatise *Bimetallism* (1897), a book the economist John Maynard Keynes (1883–1946) described as being so popular that “it remained the standard text book on the subject for students until questions in bimetallism had disappeared from the examination papers.”^114^ The typeface of *The Tides* exhibits many similarities to that used in *Bimetallism*; both books have capitalized title pages and use large, well-spaced lines of serif font in the main body, showing how George Darwin’s book was designed as an accessible publication by which Darwin’s Lowell Lectures were transferred into print.

In addition to commissioning new woodcuts and reusing blocks from earlier publications, it was necessary for Darwin and Wilson to adapt several of the electrotype plates used in the *Century Magazine*. Among these was an image based on Roberts’s photograph depicting “Nebula in Andromeda” that Darwin published in the *Century Magazine* in 1890.^115^ However, this large, square image with rounded corners did not fit the page size designated for *The Tides.* Writing to Murray, Darwin noted that “I am however dismayed to find that this block as it stands will not go on to our page.”^116^ However, Darwin outlined a solution: “if however certain margins which are not at all essential are cut off it will just go in. . .tomorrow I will show the figure to Wilson and ask him if it can be cut down without expense.”^117^ Wilson informed Darwin that it was possible to reduce the image and, to better convey this information, Darwin “enclosed a drawing of the illustration of original size, together with the portion to be cut off” (Figure 4).^118^ The process of editing the original image reflects the substantial differences between transferring single lectures into articles and a series of lectures into a book. The larger compilation put pressure on the page size, limiting the size of the illustrations. This meant Darwin had to rely on Wilson’s expertise to arrive at a middle ground in the production process, meeting Murray’s specifications for the page sizes.

**Figure 5.**
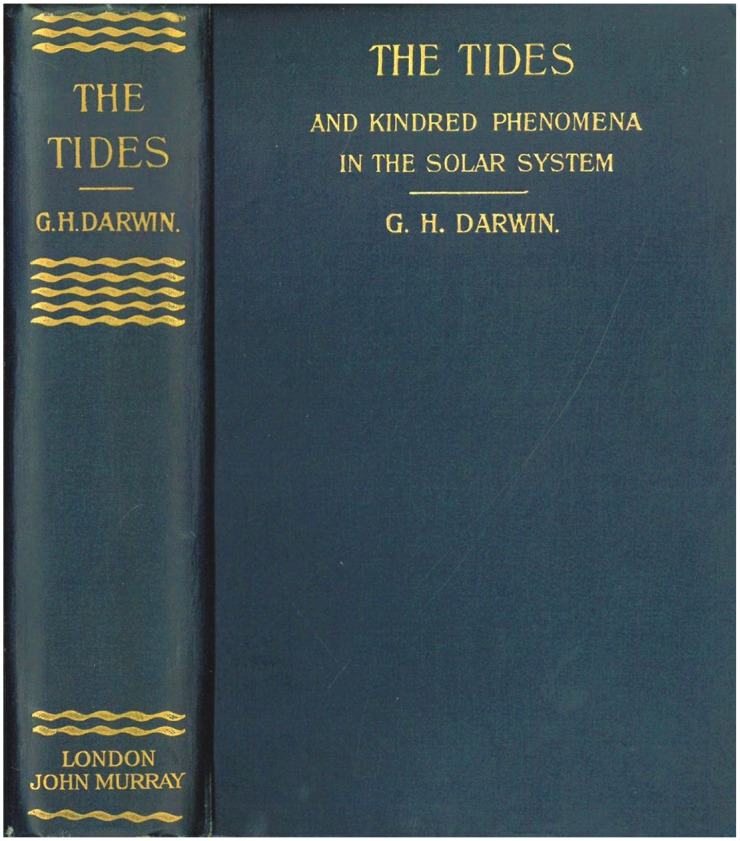
The spine and front cover of Darwin’s *The Tides* (1911). All the editions published by John Murray were decorated in a similar manner.

By September 1898, Murray had commissioned Spottiswoode and Co. to print 1,000 copies of *The Tides*. Throughout this process, the printers paid attention to Darwin’s specifications, including the drafted title page Darwin designed with his friend and neighbor, the classical scholar Richard Claverhouse Jebb (1841–1905).^119^ By the end of July, Darwin dispatched the “final proof of 120pp to Spottiswoode.”^120^ However, the binding and ornamentation of the book remained a central part of the production process that still required completion. This had to be more attractive to a broad middle-class audience than other textbooks, embodying vestiges of the spectacle evoked through public lectures. For example, earlier British Lowell Institute lecturers who based books on the content of their talks ensured these remained appealing objects to middle-class readers in Britain and America. Examples include Wallace’s *Darwinism*, published by Macmillan in Britain and the United States, a book bound in an attractive dark green cloth with gilded lettering and lines at the head and tail of the spine.^121^ Darwin had similar concerns about the binding of *The Tides*. Writing to Murray on July 31, 1898, Darwin presumed “the question of external appearance must be settled shortly. I think buckram has a good appearance, but I don’t know about its expense. I think blue would be a good colour. How about the price?”^122^ Blue buckram follows very similar conventions to Leonard Darwin’s *Bimetallism* that Murray published in the previous year. However, Darwin’s *Tides* had significantly more gilding on the spine and front board, ensuring both its practicality and status on the shelves of middle-class homes. Writing to Murray after receiving a selection of sample bindings, Darwin noted: “I like the blue pattern the better of the two for my book, but I like the way my name is put on the side better in the given sample. I also think there is too much of the gilded wavy lines on the back; three lines above & four or five below would be enough.”^123^ In comparison to books where the author is only named on the spine, Darwin’s name appears in gold lettering on both the front board and spine, where it is surrounded by wavy lines to symbolize the subject of *The Tides* (Figure 5).

## Sales, profit, and reception

Murray’s anticipation of demand for Darwin’s work was recognized by several other publishers. At the start of his course of Lowell Lectures, Darwin received a letter from a New York publisher in which the editor wrote “to express the hope that G. P. Putnam’s Sons may be afforded an opportunity of arranging with you for the publication of the volume that will probably be shaped on the material of your lectures.”^124^ However, rather than accepting Putnam’s request, Darwin chose to contract with Houghton Mifflin & Co. to publish *The Tides* in the United States and Canada, agreeing with Murray that “you will send advanced proofs, and that you will communicate with them as to the date of publication.”^125^ Houghton Mifflin & Co. planned to publish the American edition of *The Tides* in the early autumn of 1898 and suggested “for simultaneous publication of the book in England.”^126^

Murray’s prediction of high sales for the English edition rang true. Out of the 1,010 copies initially printed and marketed for sale in September 1898, Murray had only 211 on hand by the following June and only fifty-eight remained in stock by June 1900.^127^ In comparison, Houghton Mifflin & Co. were far less successful. Writing to Darwin from Boston in December 1898, the editor commented, “We regret to say that the first sales of your book have not been very large, about 350 copies. . .We hope that the English edition of the book had better success.”^128^ Low sales in North America might well have been a result of Darwin publishing several articles based on his Lowell Lectures in American magazines, meaning the content already had a broad circulation in the United States.^129^ In addition, Darwin only had a limited influence over the physical construction of the American edition. While the English edition was bound according to Darwin’s specifications in dark blue with gold wavy lines embossed on the spine, the American edition was bound in simple maroon cloth. As such, the underwhelming physical appearance of the American edition of *The Tides* might have had an impact on commercial sales, with the publisher noting that “there will be a certain regular sale of it to libraries, though the demand for a scientific book on a subject which as yet receives the attention of few students is in the nature of things somewhat limited.”^130^

A crucial reason for Houghton Mifflin & Co.’s lack of success was that Murray’s edition of *The Tides* was published and distributed in October 1898. Writing to Darwin on November 11, 1898, Augustus Lowell expressed his gratitude for a presentation copy “of your book on the Tides, which you have so kindly sent me.”^131^ Similarly, complimentary copies were distributed to those who gave Darwin permission to use their images. A week after Lowell responded, Moore wrote to thank Darwin “for the copy of your work ‘The Tides’ which I have just received from Mr. Murray and which I shall value very much.”^132^ This shows Murray’s edition was released in Britain several months before the American edition, many copies of which were not distributed until early 1899, meaning the main institutions and individuals who were going to acquire Darwin’s book in the United States and Canada already had copies of the London edition. When compared to Houghton Mifflin & Co., Murray’s success gave the premise for several new editions that Darwin adapted and corrected alongside his ongoing research.

In 1901, Murray published a second edition of *The Tides* in 505 copies, a book that sold out by 1910.^133^ This led to the heavily revised third edition appearing in 1911 that Murray published in 1,000 copies.^134^ The consistent demand for Darwin’s work from libraries, schools, and more general readers generated numerous editions that allowed Darwin to update the text alongside advances in the field he founded. In the preface to the 1911 edition, Darwin admitted that “science is always moving onwards, and books on scientific subjects need frequent revision.”^135^ These included heavy revisions to the final chapters, updating Darwin’s views on the nebulae hypothesis and the addition of a new chapter on “the figures of equilibrium of a rotating mass of fluid.”^136^ This was adapted from the content of a chapter entitled “the genesis of double stars” that Darwin contributed to *Darwin and Modern Science* (1909), a book published to celebrate the centenary of Charles Darwin’s birth and based on a public lecture series.^137^ This built on Darwin’s earlier work and that of his former doctoral student, James Hopwood Jeans, on infinitely rotating cylinders of liquid in space, leading to the theory of how, as stability diminishes, these cylinders become more pear shaped before the neck breaks to form a satellite. This has direct relevance to Darwin’s theory of the initial formation of the earth–moon system in the deep past.^138^ The necessity to continually update *The Tides* was clear evidence of its success. In the preface to the third edition, Darwin outlined that “it must generally be satisfactory to an author to find that he has succeeded in communicating to his readers some of his own interest in his subject, and the demand for a new edition of a book affords the most convincing proof of such success.”^139^ New editions of *The Tides* followed Darwin’s continued university and public lectures, for which he had lantern slides made to depict the “Section of a rotating cylinder of liquid,” images he reproduced in *Darwin and Modern Science* and transferred into the 1911 edition of *The Tides*.^140^

Darwin’s work either sold well or consistently after its initial publication in 1898, for which he received an author’s royalty of 15 percent “on the advertised price of all copies sold in England.”^141^ This is a very different author’s royalty when compared to the two-thirds profit Darwin and his siblings received from Murray for the new editions of his father’s *On the Origin of Species* (1898) and *Descent of Man* (1901).^142^ The smaller royalty margin reflects on the commercial viability of Darwin’s *Tides*. In comparison to the *Origin*, of which Murray printed 7,000 copies in 1898 and another 14,075 per printing of the new 1901 “popular impression,” George Darwin’s textbook had a more limited audience and it took Murray longer to shift the stock.^143^ This was already apparent after the 1901 edition sold out in 1909, when C. E. Lawrence wrote to Darwin from Murray’s offices to state that “Of the 500 copies printed in 1901 only about 20 remain on hand, the sale in recent years has been slow.”^144^ After the 1911 edition was published, Murray informed Darwin in the following April that “the other day you asked me how the new edition was moving; I see that we printed 1006 copies and up to the present we have by sale and presentation disposed of close to 300 copies.”^145^ Sales were hindered by Darwin’s sudden death from pancreatic cancer in 1912, meaning dispersal of *The Tides* was no longer stimulated by his public lectures and teaching in Cambridge.

After Darwin’s death, the royalties from *The Tides* were divided between three of his children, Charles Galton Darwin (1887–1962), William Robert Darwin (1894–1970), and Margaret Keynes (1890–1974).^146^ The book continued to sell at about thirty to eighty copies per year before it finally sold out in 1927.^147^ Darwin’s brother, Leonard, was appointed as his main literary executor, responsible for ensuring the transfer of the royalties and the authorization of any new editions and translations. This became a pressing issue since just before his death in 1912, George Darwin received a letter from the Spanish naval captain Leon Herrero stating, “I intend to translate your notable book The Tides (third edition) into Spanish.”^148^ Darwin granted the rights, although it was up to his brother, Leonard, and eldest son, Charles, to manage these translations. Charles permitted Herrero to preface the Spanish edition with an obituary of his father, noting in a letter to Murray that “I think the best notice of him was one which appeared in the ‘Observatory’, and I should think the translator could get all he wants from that.”^149^ In comparison to many of the translations published during Darwin’s lifetime, for which he personally approved the quality of the production, Darwin’s death meant he had limited influence over the Spanish translation. This was by no means the only foreign translation of Darwin’s work. The third edition of 1911 saw the simultaneous publication of a second German translation, the first of which came out in 1902.^150^ *The Tides* was also translated into Italian (1905) and Hungarian (1904).^151^ Writing in 1904 to Radó von Kövesligethy (1862–1934) in Budapest, Darwin communicated, “I have today received two handsomely bound copies of my book, and I feel I must thank you personally for the interest you have taken in its publication,” adding that “the book is decidedly better looking than the English edition, and I regret my inability to read your language when I turn over the pages.”^152^

The rapid initial sales of *The Tides* and its translation into four different languages resulted from its broad reception among educated society. Darwin’s respective publishers, including John Murray and Houghton Mifflin & Co., in addition to those in Germany, Italy, Hungary, and Spain, ensured the book was well advertised and reviewed in newspapers. Murray authorized the publication of a review in the *London Morning Post* in which it was suggested that Darwin’s book acted “as a stepping-stone between the primer and the advanced work,” adding that “the reader will find that, with a fair understanding of astronomy, it will not be difficult to follow the discussions on the hypotheses advanced.”^153^ This is a very similar level of praise to that Darwin’s peers gave the work. Writing in the journal *Science*, the American engineer, physicist, and mathematician Robert Simpson Woodward (1849–1924) took a similar line to the popular press, commenting that Darwin had “produced a charmingly interesting and instructive book, which may be read with profit by those who know much as well as by those who know little on the tides.”^154^ Woodward stressed that Darwin’s book served to supply a valuable general knowledge of the tides through condensing complex mathematical theories into an engaging general account.

Although the merits of Darwin’s book remained the most prominent, it did have some detractors. Writing to the editor of the *London Morning Post*, J. H. S Moxly, Chaplain of the Royal Hospital, Chelsea, wished to “warn your readers against accepting, without inquiry, the statements of even so-well a teacher as the author of the work in question,” believing that Darwin’s comments on vertical attraction were inaccurate and would be seen as obvious errors to all with “an elementary knowledge of hydrodynamics.”^155^ Other critics viewed Darwin’s work as too complex for a general audience. Writing in one brief note someone going by the pseudonym “mariner” suggested “the theory by which he [Darwin] explains the Tides is really very hard to swallow, even by a ‘simple sailor.’”^156^ This reflects the limitation of Darwin’s *Tides* to those who had access to higher levels of education – a similar audience to those who were attracted to Darwin’s lectures.

## Conclusion

Darwin’s Lowell Lectures and subsequent monograph on the tides connect the diverse content and material culture of public lectures with the complex processes used for producing a printed book at the turn of the twentieth century. Central to understanding this process is Darwin’s use of images, the content of which he transferred between the lantern slides he had projected for large audiences at the Lowell Institute through to the woodcuts and electrotype plates produced for and printed in each edition of *The Tides*. The main purpose of these images, ranging from tidal bores in eastern China to the first photographic representations of deep space, was to engage and entertain a broad, albeit an educated well-to-do, audience through the mediums of an animated public lecture and a printed book. These mediums facilitated both a temporary and a more permanent model for conveying the complex ideas Darwin developed to found the field of geophysics by the turn of the twentieth century. As such, Darwin presents a key insight into the processes used by numerous public lecturers when converting talks into monographs, which saw a surge in popularity at the turn of the twentieth century.

The relative success of *The Tides* was connected to Darwin’s consistent public, academic, and university lectures. As we have seen, many of Darwin’s public lectures were given for the Birmingham and Midland Institute, the Gilchrist Trust, the Royal Institution, and, of course, the Lowell Institute, who all catered for well-educated middle-class audiences who also had the income to purchase a monograph based on these talks. A gilt spine would have been an appealing decoration on a bookshelf in a middle-class home. In addition to giving numerous talks in Britain and the United States, Darwin lectured for scientific and public audiences at events such as the International Geodetic Congresses in Stuttgart (1898), Paris (1900), Copenhagen (1903), Budapest (1906), and Cambridge (1909), where Darwin and his wife Maud entertained numerous delegates at Newnham Grange.^157^ In 1905, Darwin traveled to South Africa as the president of the British Association for the Advancement of Science (BAAS), a tour launched to solidify British rule in the region in the immediate aftermath of the Boer War.^158^ Darwin’s diary gives numerous descriptions of his own and his colleagues’ public and academic lectures as he journeyed overland between the Cape and Cairo. For example, Darwin gave the opening address for the BAAS on Tuesday, August 15, 1905, and when visiting the Kimberley diamond mine on September 5, Darwin noted that “At half-past eight in the evening Sir William Crookes gave a lecture on diamonds. The lecture was an admirable one and his experiments succeeded to perfection, but he made the enormous mistake of lecturing for two hours so that everybody was weary at the end.”^159^ Similarly, Darwin described numerous garden parties and civic events at which he gave brief public addresses, such as the opening of the Bulawayo Museum.^160^

Darwin’s continued public and academic lecture tours acted as an international advertising campaign for *The Tides*. This was combined with his public lectures around Britain and consistent university teaching, which generated sales from the British public and his students at Cambridge. The lantern slides Darwin commissioned accompanied him on these trips, acting as central illustrative aids for his talks while also serving as promotional material for his book. As Darwin’s research progressed, so too did the content of his lectures and the number of slides he showed – something also represented by the increased content formed from text and images in each new edition of *The Tides*. After Darwin’s death from pancreatic cancer in 1912, Murray’s accounts show that sales of *The Tides* slumped to between fifty and eighty copies per year, with the final eighteen copies selling out in 1927.^161^

The close material connections between Darwin’s lectures and *The Tides* show how these approaches to conveying information developed alongside one another. Darwin’s lectures and book on tides present a very different relationship between print and public talks when compared with previous speakers at the Lowell Institute and other British lecturers in the United States such as Tyndall, Richard Proctor, and Peter Kropotkin, who relied on newsprint to promote their talks and ideas to mass audiences. In comparison, Darwin’s interest in producing a book published in multiple editions allowed for the continued public promotion of his research as it progressed, showing how Darwin’s academic research was intimately bound up with his attempts to convey complex ideas to broader audiences. It provides a framework for the practices used by other British Lowell Institute lecturers who published books based on their talks, perhaps the most notable being Wallace’s *Darwinism*, which was published in three editions and several different languages during his lifetime. Darwin’s theories on tides and the evolution of satellites and the wider solar system remained valid for much of the twentieth century. A renewed interest in Darwin’s field by the early 1960s encouraged the San Francisco publisher W. H. Freeman to publish yet another edition of *The Tides* – a paperback aimed at a broader audience. In the preface, the editor paid tribute “to the remarkable progress achieved by Sir George Darwin in a field essentially founded by him as it is to the lack of progress ever since.”^162^

## Notes

1. “Prof. Darwin Comes Today,” *Boston Herald*, October 13, 1897, p.4.

2. For the Darwin dynasty, see Noel Annan, *The Dons: Mentors, Eccentrics and Geniuses* (London: HarperCollins, 1999). For a recent account of the Huxley dynasty, see Alison Bashford, *The Huxleys: An Intimate History of Evolution* (Chicago, IL: University of Chicago Press, 2022).

3. Edward Everett, *A Memoir of Mr. John Lowell, Jun. Delivered as the Introduction to the Lectures on his Foundation in the Odeon, 31st December 1839; Repeated at Marlborough Chapel, 2nd January, 1840* (Boston, MA: Charles C. Little, 1840), pp.65–6; Chaim M. Rosenberg, *John Lowell Jr. and His Institute* (Lanham, MD: Lexington Books, 2021), pp.xi–xii.

4. “Prof. Darwin Comes Today,” *Boston Herald*, October 13, 1897, p.4; Diarmid A. Finnigan, *The Voice of Science: British Scientists on the Lecture Circuit in Gilded Age America* (Pittsburgh, PA: University of Pittsburgh Press, 2021), pp.16–17. See page 22 for a full list of British lecturers at the Lowell Institute in the late nineteenth-century.

5. Finnigan, *Voice of Science*, p.17 (note 4).

6. Margaret W. Rossiter, “Benjamin Silliman and the Lowell Institute: The Popularisation of Science in Nineteenth-Century America,” *New England Quarterly* 44 (1971): 602–26.

7. Donald M. Scott, “The Popular Lecture and the Creation of a Public in Mid-Nineteenth-Century America,” *Journal of American History* 66 (1980): 791–809, 803.

8. Bernard Lightman, *Victorian Popularisers of Science: Designing Nature for New Audiences* (Chicago, IL: University of Chicago Press, 2007), pp.viii–ix.

9. Peter J. Bowler, *Science for All: The Popularization of Science in Early Twentieth-Century Britain* (Chicago, IL: University of Chicago Press, 2009).

10. Ibid., pp.1–2, 10.

11. Joshua Nall, *News from Mars: Mass Media and the Forging of a New Astronomy, 1860–1910* (Pittsburgh, PA: University of Pittsburgh Press, 2019), pp.135–59.

12. George H. Darwin, *The Tides and Kindred Phenomena of the Solar System* (London: John Murray, 1898).

13. For more detail on the material culture of scientific lecturing, see Bernard Lightman, “Lecturing in the Spatial Economy of Science,” in Aileen Fyfe and Bernard Lightman (eds.), *Science in the Marketplace: Nineteenth-Century Sites and Experiences* (Chicago, IL: University of Chicago Press, 2007), pp.97–134; Emly Hayes, “Slidescapes: Three Royal Geographical Society Lectures by Vaughan Cornish,” *Early Popular Visual Culture* 17 (2019): 71–88; Emily Hayes, “Geographical Light: The Magic Lantern, the Reform of the Royal Geographical Society and the Professionalisation of Geography c. 1885–1894,” *Journal of Historical Geography* 62 (2018): 24–36; Richard Crangle, Mervyn Heard, and Ine van Dooren (eds.), *Realms of Light: Uses and Perceptions of the Magic Lantern from the 17th to the 21st Century* (London: The Magic Lantern Society, 2005).

14. See Sabine Lenk and Natalija Majsova (eds.), *Faith in a Beam of Light: Magic Lantern and Belief in Western Europe, 1860–1940* (Turnhout, Belgium: Brepols, 2022).

15. Noel Annan, “The Intellectual Aristocracy,” in J. H. Plumb (ed.), *Studies in Social History: A Tribute to G. M. Trevelyan* (London: Longmans, 1955), pp.241–87; Bernard Lightman, “Darwin and the Popularization of Evolution,” *Notes and Records of the Royal Society* 64 (2010): 5–24.

16. James A. Secord, “Darwin on Paper” (Text of a Lecture Given at Universidad Nacional Autónoma de México, Mexico City, November 24, 2022).

17. This property is now part of Darwin College, Cambridge.

18. For more on Maud’s prominent social position in Cambridge, see Frances Spalding, *Gwen Raverat: Friends, Family and Affections* (London: Pimlico, 2004).

19. Rosenberg, *John Lowell*, pp.213–37 (note 3).

20. Letter from Augustus Lowell to George Darwin, April 22, 1896, Cambridge University Library, Cambridge (hereafter CUL), DAR 251: 4306.

21. Letter from George Darwin to Augustus Lowell, May 10, 1896, “Lowell Institute Records, circa 1837–1943,” Houghton Library, Harvard University, 54M-206.

22. Quoted in Everett, *Memoir*, p.66 (note 3).

23. Margaret Keynes, *A House by the River: Newnham Grange to Darwin College* (Cambridge: Private Press, 1984), p.165.

24. George Howard Darwin, “On the Influence of Geological Changes on the Earth’s Axis of Rotation,” *Philosophical Transactions* 167 (1877): 271–312; David Kushner, “George Howard Darwin and the British School of Geophysics,” *Osiris* 8 (1993): 196–224, 201.

25. Ibid., 202. Darwin was building on several earlier theories to make this proposition; see Miguel Ohnesorge, “Pluralizing Measurement: Physical Geodesy’s Measurement Problem and Its Resolution,” *Studies in History and Philosophy of Science* 96 (2022): 51–67, 57–62.

26. Kushner, “George Howard Darwin,” 201 (note 24).

27. A. Maton (Cambridge University Press) to Darwin, May 22, 1891, CUL, DAR 251: 3908.

28. Numerous tide tables Darwin received are held by CUL, MS Add. 5749. See Keynes, *A House by the River*, p.163 (note 23).

29. David Kushner, “Darwin, Sir George Howard (1845–1912), Mathematician and Geophysicist,” *Oxford Dictionary of National Biography*, September 23, 2004, <www.oxforddnb.com/view/10.1093/ref:odnb/9780198614128.001.0001/odnb-9780198614128-e-32718> (February 2, 2023).

30. M. Foster to Darwin, June 10, 1896, CUL, DAR 251: 4332. For computers in the period, see Simon Schaffer, “Babbage’s Intelligence: Calculating Engines and the Factory System,” *Critical Inquiry* 21 (1994): 203–27.

31. Lowell to Darwin, March 4, 1897, CUL, DAR 251: 1589.

32. “Some Peculiarities at the Lowell Institute,” CUL, DAR 251: 1589.

33. Jeremy Brooker, “A Lecture in Locust Street: Morton, Tyndall, and the Construction of Scientific Reputation,” in Carin Berkowitz and Bernard Lightman (eds.), *Science Museums in Transition: Cultures of Display in Nineteenth-Century Britain and America* (Pittsburgh, PA: University of Pittsburgh Press, 2017), pp.111–38, p.132.

34. “Professor Tyndall’s Lecture: His First Appearance in Boston: A Large and Interested Audience,” *Boston Evening Journal*, October 16, 1872, p.1.

35. “Lowell Institute Lectures,” CUL DAR 251: 1589.

36. Ahti-Veikko Pietarinen (ed.), *Charles S. Peirce: The Logic of the Future: The 1903 Lowell Lectures* (Berlin, Germany and Boston, MA: De Gruyter, 2021), p.38.

37. “Lowell Institute Lectures,” CUL DAR 251: 1589.

38. Darwin to Lowell, March 17, 1897, “Lowell Institute Records, circa 1837–1943,” Houghton Library, Harvard University, 54M-206.

39. Darwin to Lowell, February 14, 1897, “Lowell Institute Records, circa 1837–1943,” Houghton Library, Harvard University, 54M-206.

40. The rule of only showing slides at the end was still enforced in 1903. See Pietarinen, *Charles S. Peirce*, p.38 (note 36).

41. Quoted in Lightman, “Lecturing in the Spatial Economy of Science,” p.97 (note 13).

42. Ibid., pp.99–103.

43. “Prof. Darwin Comes Today. The Famous Scientist Will Lecture Friday Night,” *Boston Herald*, October 13, 1897, p.4.

44. Lowell Institute Scrapbook, “George H. Darwin,” “Lowell Institute records, circa 1837-1943,” Houghton Library, Harvard University, 54M-206.

45. “Knowledge about Tides,” *Boston Herald*, October 16, 1897, p.6

46. Francis Darwin, *Memoir of Sir George Darwin* in *Scientific Papers by Sir George Howard Darwin*, Vol. V (Cambridge: Cambridge University Press, 1916), p.xxvii. Numerous invitations to lecture for these institutes can be found in Darwin’s correspondence. Many are followed by letters concerning the specific arrangements for travel and showing lantern slides.

47. Lightman, “Lecturing in the Spatial Economy of Science,” pp.97–9 (note 13); Mark Butterworth, “A Lantern Tour of Star-Land: The Astronomer Robert Ball and his Magic Lantern Lectures,” in Richard Crangle, Mervyn Heard, and Ine van Dooren (eds.), *Realms of Light: Uses and Perceptions of the Magic Lantern from the 17th to the 21st Century: An Illustrated Collection of Essays by 27 Authors from Six Countries* (London: Magic Lantern Society, 2005), pp. 162–73.

48. “Knowledge on Tides,” *Boston Herald*, October 23, 1897, p.12.

49. “Knowledge about Tides,” *Boston Herald*, October 16, 1897, p.6.

50. Lowell Institute Scrapbook, “George H. Darwin,” “Lowell Institute Records, circa 1837–1943,” Houghton Library, Harvard University, 54M-206.

51. Hayes, “Geographical Light,” 29 (note 13).

52. Butterworth, “A Lantern Tour of Star-Land,” p.172 (note 47).

53. Ibid., p.171.

54. William Usborne Moore, *Further Report on the Bore of the Tsien-Tang-Kiang* (London: Hydrographic Office, 1893).

55. For Britain’s “sphere of influence” in China, see T. G. Otte, *The China Question: Great Power Rivalry and British Isolation, 1894–1905* (Oxford: Oxford University Press, 2007).

56. Moore, *Further Report* (note 54).

57. Kushner, “George Howard Darwin,” 206 (note 24).

58. George Darwin’s lantern slides are now held by Darwin College, Cambridge.

59. George H. Darwin, *The Tides and Kindred Phenomena of the Solar System*, 3rd ed. (London: John Murray, 1911), pp.64–8.

60. Ibid., p.62.

61. “Can’t Be Done: Prof. Darwin Says That Tides Are Not Available for Generating Power,” *Boston Evening Journal*, October 23, 1897, p.1.

62. J. B. S. Haldane, *The Last Judgement: A Scientist’s Vision of the Future of Man* (New York, NY and London: Harper & Brothers, 1927), p.11.

63. Mark B. Adams, “Last Judgement: The Visionary Biology of J. B. S. Haldane,” *Journal of the History of Biology* 33 (2000): 457–91, 464.

64. Darwin, *The Tides* (1911), pp.70–1 (note 59).

65. Ibid., pp.71–3.

66. Ibid., p.73.

67. Although many of these reports were offered for sale, their lack of commercial viability placed severe limitations on the volume of copies produced and their distribution; see Oz Frankel, *States of Inquiry: Social Investigation and Print Culture in Nineteenth-Century Britain and the United States* (Baltimore, MD: Johns Hopkins University Press, 2006), p.19.

68. Darwin, *The Tides* (1911), p.74 (note 59).

69. Keynes, *A House by the River*, pp.163–4 (note 23).

70. Darwin to “Mr Pierce, Janitor of the Huntington Hall,” November 21, 1897, “Lowell Institute Records, circa 1837–1943,” Houghton Library, Harvard University, 54M-206.

71. “Tides in the Earth,” *Boston Herald*, November 10, 1897, p.6.

72. Darwin, *The Tides* (1911), pp.115–16 (note 59).

73. “Prof. Darwin’s Second Lecture,” *Boston Herald*, October 17, 1897, p.6.

74. Darwin, *The Tides* (1911), p.116 (note 59).

75. Ibid., p.124.

76. Ibid., p.125.

77. Lantern slide labeled “Neb. Andromeda by Isaac Roberts 19 Decr. 1888 [in the hand of Isaac Roberts],” Darwin College, Cambridge.

78. Frederick Bramwell to Darwin, December 11, 1888, CUL, DAR 251: 3270.

79. H. H. Turner, quoted in Robert W. Smith, “The ‘Great Plan of the Visible Universe’: William Huggins, Evolutionary Naturalism and the Nature of the Nebulae,” in Bernard Lightman and Michael S. Reidy (eds.), *The Age of Scientific Naturalism: Tyndall and His Contemporaries* (Oxon: Pickering and Chatto, 2014), pp.113–36, p.132.

80. Lightman, *Victorian Popularisers of Science*, p.482 (note 8).

81. Arthur Nichols to Darwin, January 31, 1889, CUL, DAR 251: 3369.

82. Isaac Roberts to Darwin, December 4, 1888, CUL, DAR 251: 3379.

83. Roberts refers to one of the earliest depictions of Andromeda, published in George P. Bond, “An Account of the Nebula Andromeda,” *Memoirs of the American Academy of the Arts and Sciences* 3 (1848): 75–86.

84. Roberts to Darwin, December 4, 1888, CUL, DAR 251: 3379.

85. Roberts to Darwin, December 19, 1888, CUL, DAR 251: 3380.

86. Darwin [DRAFT] to William Watson Gilder, February 7, 1889, CUL, DAR 251: 3292.

87. For Laplace’s nebula hypothesis, see: Ronald L. Numbers, *Creation by Natural Law: Laplace’s Nebula Hypothesis in American Thought* (Seattle, WA and London: University of Washington Press, 1977); Stephen G. Bush, “The Nebula Hypothesis and the Evolutionary Worldview,” *History of Science* 25 (1987): 245–78.

88. Darwin, *The Tides* (1911), p.407 (note 59).

89. Ibid., pp.409–10.

90. Roberts to Darwin, January 8, 1889, CUL, DAR 251: 3381.

91. Darwin, *The Tides*, p.413 (note 12).

92. Paxton Porter to Darwin, September 28, 1887, CUL, DAR 251: 2955.

93. Charles Edward Reade to Darwin, January 28, 1889, CUL, DAR 251:3382.

94. Gilder to Darwin, February 20, 1889, CUL, DAR 251: 3293.

95. Roberts to Darwin, February 5, 1889, CUL, DAR 251: 3393.

96. Darwin to Lowell, March 17, 1897, “Lowell Institute Records, circa 1837–1943,” Houghton Library, Harvard University, 54M-206. These concerns about the intellectual property of information conveyed in public lectures had been apparent since the 1830s. See Jonathan Topham, *Reading the Book of Nature: How Eight Best Sellers Reconnected Christianity and the Sciences on the Eve of the Victorian Age* (Chicago, IL: University of Chicago Press, 2022), p.101.

97. For British scientists’ problems with copyright at the Lowell Lectures, see Finnigan, *The Voice of Science*, p.17 (note 4).

98. Darwin, *The Tides* (1911), p.ix (note 59).

99. Charles H. Smith and Megan Derr (eds.), A. R. Wallace, *Alfred Russel Wallace’s 1886–1887 Travel Diary: The North American Lecture Tour* (Manchester, UK: Siri Scientific Publications, 2013), p.8; Alfred Russel Wallace, *Darwinism: An Exposition of the Theory of Natural Selection, with Some of Its Applications* (London: Macmillan, 1889).

100. John Murray to Darwin, December 23, 1897, CUL, DAR 251: 4438.

101. George Darwin to John Murray, May 1, 1882, National Library of Scotland, Edinburgh, John Murray Archive (hereafter NLS, JMA), “Letters to John Murray, publishers, of George Howard Darwin, with related letters,” MS.40313, f. 1.

102. Ledger/Account Book, NLS, JMA, pm MS. 42735, f. 106.

103. Charles Darwin, *The Movements and Habits of Climbing Plants* (London: John Murray, 1875), p.v. George Darwin’s original illustrations for this book can be found at “NS I Chap 3-97 Drawings, 1867-01-01-1867-01-31,” CUL, MS DAR 49: 113–15.

104. Darwin to Gilder, February 7, 1889, CUL, DAR 251: 3392.

105. Darwin to John Murray, January 21, 1898, NLS, JMA, “Letters to John Murray, Publishers, of George Howard Darwin, with related letters,” MS.40313, f. 21.

106. Susannah Gibson, *The Spirit of Inquiry: How One Extraordinary Society Shaped Modern Science* (Oxford: Oxford University Press, 2019), pp.109–10.

107. David McKittrick, *A History of Cambridge University Press: Vol. 3, New Worlds for Learning, 1873–1972* (Cambridge: Cambridge University Press, 2004), pp.6–7.

108. Ibid., p.174.

109. “Agreement between Edwin Wilson and Cambridge University for the Sale to the University of Machinery, Stock, Implements, and Utensils in the Mill Lane Premises (Includes Book Debts), 1913,” CUL, UA/PRESS 1/1/2/28.

110. Roberts did not publish an image of Andromeda until 1899. See Isaac Roberts, *Photographs of Stars, Star-Clusters and Nebulae, Together with Records of Results Obtained in the Pursuit of Celestial Photography*, Vol. II (London: Knowledge Office, 1899).

111. Darwin to Murray, January 21, 1898, NLS, JMA, “Letters to John Murray, Publishers, of George Howard Darwin, with Related Letters,” MS.40313, f. 22.

112. Edwin Wilson to John Murray, March 2, 1898, NLS, JMA, “Letters to John Murray, Publishers, of George Howard Darwin, with Related Letters,” MS.40313, f. 92; Darwin to John Murray, March 22, 1898, NLS, JMA, “Letters to John Murray, Publishers, of George Howard Darwin, with Related Letters,” MS.40313, f. 29.

113. Darwin to Murray, March 17, 1898, NLS, JMA, “Letters to John Murray, Publishers, of George Howard Darwin, with Related Letters,” MS.40313, f. 98.

114. Leonard Darwin, *Bimetallism: A Summary and Examination of the Arguments for and against a Bimetallic System of Currency* (London: John Murray, 1897); John Maynard Keynes, “Obituary: Leonard Darwin (1850–1943),” *The Economic Journal* 53 (1943): 438–50, 438.

115. George H. Darwin, “On Meteorites and the History of Stellar Systems,” *Century Magazine* 40 (1890): 858–65, 865.

116. Darwin to Murray, April 27, 1898, NLS, JMA, “Letters to John Murray, Publishers, of George Howard Darwin, with Related Letters,” MS.40313, f. 34.

117. Ibid.

118. Darwin to Murray, April 28, 1898, NLS, JMA, “Letters to John Murray, Publishers, of George Howard Darwin, with Related Letters,” MS.40313, f. 36–7.

119. Darwin to Murray, May 1, 1898, NLS, JMA, “Letters to John Murray, Publishers, of George Howard Darwin, with Related Letters,” MS.40313, f. 39.

120. Ibid., f. 39.

121. Examples of the British and American editions of *Darwinism* in their original bindings can be found in the Whipple Library, Cambridge, at shelf marks:STORE 139:39; and STORE 222:28 for the American edition.

122. Darwin to Murray, July 31, 1898, NLS, JMA, “Letters to John Murray, Publishers, of George Howard Darwin, with Related Letters,” MS.40313, f. 47.

123. Darwin to Murray, August 11, 1898, NLS, JMA, “Letters to John Murray, Publishers, of George Howard Darwin, with Related Letters,” MS.40313, f. 49.

124. G. W. Putnam to Darwin, October 25, 1897, CUL, DAR 251: 4444.

125. Darwin to Murray, February 18, 1898, NLS, JMA, “Letters to John Murray, Publishers, of George Howard Darwin, with Related Letters,” MS.40313, f. 23.

126. A. S. Watt (Houghton Mifflin & Co., London office) to Darwin, February 17, 1898, CUL, DAR 251: 4426.

127. Ledger/Account Book, NLS, JMA, pm MS. 42735, f. 225.

128. Houghton Mifflin & Co to Darwin, December 14, 1898, CUL, DAR 251: 4541.

129. See, for example, George H. Darwin, “Bores,” *Century Illustrated Magazine* 56 (1898): 898–904.

130. Houghton Mifflin & Co to Darwin, December 14, 1898, CUL, DAR 251: 4541.

131. Lowell to Darwin, November 11, 1898, CUL, DAR 251: 4546.

132. Moore to Darwin, November 18, 1898, CUL, DAR 251: 4584. On the half-title of the copy of *The Tides* Darwin ordered Murray to send, Moore has written, “W. Usborne Moore, from the author 1898.” This book was sold by Hackenberg Booksellers, El Cerrito, CA, on April 24, 2023, and is now in a private collection.

133. Ledger/Account Book, NLS, JMA, pm MS. 42737, f. 214.

134. Ledger/Account Book, NLS, JMA, pm MS. 42741, f. 77.

135. Darwin, *The Tides* (1911), p.[5] (note 59).

136. Ibid.

137. Gibson, *The Spirit of Inquiry*, p.109 (note 106).

138. George H. Darwin, “Figures of Equilibrium of Rotating Masses of Fluid,” *Philosophical Transactions* 178 (1887): 379–428; James H. Jeans, “The Stability of a Spherical Nebula,” *Philosophical Transactions* 199 (1902): 312–20; George H. Darwin, “On the Figure and Stability of a Liquid Satellite,” *Philosophical Transactions of the Royal Society of London. Series A, Containing Papers of a Mathematical or Physical Character* 206 (1906): 161–248; George H. Darwin, “Further Consideration of the Stability of the Pear-Shaped Figure of a Rotating Mass of Liquid,” *Philosophical Transactions of the Royal Society of London. Series A, Containing Papers of a Mathematical or Physical Character* 208 (1908): 1–19.

139. Darwin, *The Tides* (1911), p.[5] (note 59).

140. George H. Darwin, “Genesis of Double Stars,” in A. C. Seward (ed.), *Darwin and Modern Science* (Cambridge: Cambridge University Press, 1909), pp.543–64, p.553; Darwin, *The Tides* (1911), p.375 (note 59); George Darwin lantern slide collection, Darwin College, Cambridge. See also John Clay (Cambridge University Press) to John Murray, February 19, 1911, NLS, JMA, “Book File,” for *The Tides*. Clay states to Murray, “I would be pleased to supply electros of any figures which you consider suitable” for reproduction in *The Tides* (1911) from books published with Cambridge University Press.

141. “Statement of Mr. Murray’s practice in making up accounts to authors for works on which a royalty is paid to the author,” January 13, 1911, NLS, JMA, “Book File,” for *The Tides*.

142. Ledger/Account Book, NLS, JMA, pm MS. 42735, fols. 105, 112.

143. Ledger/Account Book, NLS, JMA, pm MS.42735, fols. 105–6.

144. C. E. Lawrence to Darwin, September 22, 1909 [transfer copy], NLS, JMA, “Book File,” for *The Tides*.

145. Murray to Darwin, April 9, 1912 [transfer copy], NLS, JMA, “Book File,” for *The Tides*.

146. Executors of Darwin’s estate to John Murray, January 23, 1913, NLS, JMA, “Book File,” for *The Tides*.

147. Ledgers/Account Books, NLS, JMA, pm MS. 42741, f. 77; pm MS.42742, fols. 245, 246.

148. Leon Herrero to Darwin, September 24, 1912, NLS, JMA, “Book File,” for *The Tides*. George H. Darwin, *Las Marcas y Fenómenos de la Misma Naturaleza en el Sistema Solar*, trans. by Leon Herrero (Ferol: El Correo Gallego, 1914).

149. Charles Galton Darwin to John Murray, September 5, 1913, NLS, JMA, “Book File,” for *The Tides*. George H. Darwin.

150. George H. Darwin, *Ebbe und Flut sowie verwandte Erscheinungen im Sonnensystem. Autorisierte deutsche Ausgabe nach der zweiten englischen Auflage von Agnes Pockels. Mit einem Einführungswort von Georg von Neumayer* (Leipzig, Germany: Teubner, 1902).

151. George H. Darwin, *A Tengerjárás és Rokontünemények Naprendszerünkben. Irta – a csillágaszat tanára a cambridgei egyetemen. Fordította Dr. Kövesligethy Radó egyetemi tanár. Az eredetivel összehasonlította Dr. B. Eötvös Loránd egyetemi tanár. Ötvenkét rajzzal* (Budapest, Hungary: Természettudományi Társulat, 1904).

152. Darwin to Kövesligethy, March 18, 1904, CUL, Add. 8812/90.

153. “The Tides,” *London Morning Post*, January 10, 1899, p.3.

154. Robert Simpson Woodward, “Review of *The Tides and Kindred Phenomena in the Solar System*. By George Howard Darwin. Boston and New York, Houghton Mifflin & Co. 1898. Pp.xviii + 378,” *Science* 8 (1898): 911–12.

155. J. H. S. Moxly, “Professor Darwin on the Tides,” *London Morning Post*, Friday January 13, 1899, p.2; J. H. S. Moxly, “Professor Darwin on the Tides,” *London Morning Post*, Monday January 23, 1899, p.3.

156. “Mariner,” “Professor Darwin on Tides,” *London Morning Post*, Saturday January 28, 1899, p.3.

157. Keynes, *A House by the River*, p.157 (note 23).

158. Kushner, “George Howard Darwin,” 197 (note 24); Saul Dubow, *A Commonwealth of Knowledge: Science, Sensibility, and White South Africa, 1820–2000* (Oxford: Oxford University Press, 2006), p.173.

159. George Howard Darwin, “Diary of Journey to South Africa with the British Association. 1905,” CUL, MS Add. 8166, f. 25.

160. Ibid., f. 28.

161. Ledgers/Account Books, NLS, JMA, pm MS. 42742, fols. 245–6.

162. George Howard Darwin, *The Tides and Kindred Phenomena of the Solar System* (San Francisco, CA: W. H. Freeman, 1962), p.ix.

